# Evidence for active upper mantle flow in the Atlantic and Indo-Australian realms since the Upper Jurassic from hiatus maps and spreading rate changes

**DOI:** 10.1098/rspa.2021.0764

**Published:** 2022-06-29

**Authors:** Berta Vilacís, Jorge N. Hayek, Ingo L. Stotz, Hans-Peter Bunge, Anke M. Friedrich, Sara Carena, Stuart Clark

**Affiliations:** ^1^ Department of Earth and Environmental Sciences, Ludwig-Maximilians-Universität München, Theresienstraße 41 and Luisenstraße 37, Munich 80333 Germany; ^2^ University of New South Wales Sydney, Minerals and Energy Res. Eng., Kensington, New South Wales 2052, Australia

**Keywords:** dynamic topography, hiatus, oceanic spreading rates, Poiseuille flow, mantle convection, global geodynamics

## Abstract

Histories of large-scale horizontal and vertical lithosphere motion hold important information on mantle convection. Here, we compare continent-scale hiatus maps as a proxy for mantle flow induced dynamic topography and plate motion variations in the Atlantic and Indo-Australian realms since the Upper Jurassic, finding they frequently correlate, except when plate boundary forces may play a significant role. This correlation agrees with descriptions of asthenosphere flow beneath tectonic plates in terms of Poiseuille/Couette flow, as it explicitly relates plate motion changes, induced by evolving basal shear forces, to non-isostatic vertical motion of the lithosphere. Our analysis reveals a timescale, on the order of a geological series, between the occurrence of continent-scale hiatus and plate motion changes. This is consistent with the presence of a weak upper mantle. It also shows a spatial scale for interregional hiatus, on the order of 2000–3000 km in diameter, which can be linked by fluid dynamic analysis to active upper mantle flow regions. Our results suggest future studies should pursue large-scale horizontal and vertical lithosphere motion in combination, to track the expressions of past mantle flow. Such studies would provide powerful constraints for adjoint-based geodynamic inverse models of past mantle convection.

## 1. Introduction

An enduring theme in mantle flow studies is the existence of an asthenosphere (see [1] for a review). The presence of this layer was advocated by Barrell [2] to allow for isostatic movement, and by Chase [3] to lubricate plate motion. Modern evidence for a mechanically weak layer in the uppermost mantle beneath the lithosphere comes from a variety of observations. They include studies of the geoid (e.g. [4]), post-glacial rebound (e.g. [5]), lake loading (e.g. [6]), oceanic intraplate seismicity [7], ocean ridge bathymetry [8] and seismic anisotropy [9,10]. Fluid dynamic investigations employing numerical and analytic modelling techniques (e.g. [11–13]) agree that high material mobility in the asthenosphere is essential to promote the long-wavelength character of mantle flow observed on Earth. Three-dimensional spherical Earth models that combine an asthenosphere and a plastic yield stress to allow localized weakening of the cold upper thermal boundary layer result in a distinctly plate tectonic style of convection [14].

**Figure 1.**
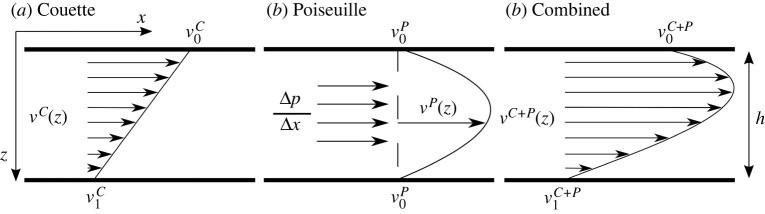
Schematic of (*a*) Couette flow, described a linear profile of shear driven fluid motion between two surfaces where one moves tangentially relative to the other, and seen as proxy for passive asthenosphere flow driven by motion of the overlying plate, (*b*) Poiseuille flow, described by a parabolic profile, driven by a lateral pressure gradient, and seen as a proxy for active asthenosphere flow capable of driving the overlying plate, and (*c*) superposition of both.

Morgan and colleagues [15,16] linked the asthenosphere to mantle plumes, proposing an asthenosphere fed actively by hot upwellings. This and their subsequent work [17,18] explored the resulting upper mantle flow in terms of a simple model, where material flux is driven horizontally by lateral pressure variations, in order to explain various observations related to ocean bathymetry, heat flow and mantle geochemistry. A series of papers by Höink *et al.* [19–22] extended this approach. They formulated mantle convection models explicitly in the context of so-called Poiseuille/Couette flow (figure 1). An important finding is that lateral pressure variations in the asthenosphere are not tied exclusively to the influx of plume material, but occur also for convection heated purely from within. Poiseuille/Couette flow is thus an intrinsic property of material flowing in a low viscosity asthenosphere channel beneath mobile tectonic plates.

Asthenosphere flow driven by high- and low-pressure regions is a powerful concept, because it connects mantle flow to geologic observations in a testable manner. In particular, it explicitly relates temporal changes in horizontal plate motion, i.e. oceanic spreading rate variations driven by evolving basal shear stresses, to non-isostatic vertical motion of the lithosphere. The latter is a form of topography maintained dynamically by mantle convection. It was termed ‘dynamic topography’ by Hager *et al.* [23] more than 30 years ago (see [24] for a review).

Current plate motions are well mapped [25]. But histories of plate motion are becoming better known. Initially documented for the Cenozoic [26], global plate motion reconstructions are now available for times going back to the Mesozoic (see [27] for a review). Progress is also underway in mapping the magnetization of some ocean floor regions in great detail, permitting plate motion reconstructions at temporal resolutions of ∼1 million years (Myrs) or less (e.g. [28,29]) when mitigating for finite-rotation noise [30]. These efforts reveal that it often takes but a few Myrs for plates to change their motions. The variations may be due to changes in plate boundary forces [31] and/or basal shear stresses [32]. They provide important observations for geodynamic interpretations (see [33] for a review).

Less is known about current dynamic topography, at least outside the oceanic realm [34]. Dynamic topography for continents is difficult to map, because one needs to separate it from topography in isostatic support [35,36]. But histories of dynamic topography in the continents are beginning to emerge, because the transient nature of dynamic topography leaves geologic evidence in sedimentary archives [37]. This approach was pioneered for regions that underwent periods of low dynamic topography, such as the *Cretaceous Interior Seaway* of North America (e.g. [38,39]), where the associated surface depressions created accommodation space to preserve sediments.

It is more difficult to map the stratigraphic expression of dynamic topography highs. The associated elevated topography creates erosional/non-depositional environments expressed as time gaps in the geologic record. The resulting discontinuity surfaces in sedimentary archives are known as non conformities and unconformities (see [40] for a review). They preserve time missing (hiatus) from the geological record [41]. To this end, an approach of hiatus-area mapping was introduced by Friedrich *et al.* [41] and Friedrich [42]. It visualizes interregional-scale unconformities because, at continental scales, what is normally perceived as a lack of data (material eroded or not deposited) becomes part of the dynamic topography signal. The method has been applied to map the spatio-temporal patterns of conformable and unconformable geological contacts across Europe [43], Africa [44] and the Atlantic realm and Australia since the Upper Jurassic [45,46]. An important finding is that significant differences exist in the spatial extent of hiatus area across and between continents at the timescale of geologic series, that is ten to a few tens of Myrs (see definition of series as a unit of chronostratigraphy in the chronostratigraphic chart [47,48]). This is considerably smaller than the mantle transit time, which as the convective timescale is about 100–200 Myrs [33] for Earth’s mantle. The difference between the convective timescale and the timescale for topography in convective support suggests vigorous upper mantle flow, as illustrated by geodynamic kernels (see [49] for a review).

Pressure driven asthenosphere flow explicitly relates plate motion changes, induced by evolving basal shear forces, to non-isostatic vertical motion of the lithosphere, as noted before. Such correlations were reported for the South [32] and North [43] Atlantic. Here, we extend this observational geodynamic approach. We take the hiatus maps of Hayek *et al.* [45,46], with an updated dataset for Australia following the approach of Carena *et al.* [44], as proxy for mantle flow induced vertical lithosphere motion and compare them systematically to horizontal plate motion variations deduced from a past plate motion model based on the dataset of Müller *et al.* [27]. We organize our paper as follows: §2 and 3 provide brief summaries of plate motion variations and hiatus surfaces for the Atlantic and Indo-Australian realm. We study these regions to account for ocean basins with and without significant subduction activity, while our choice of the Upper Jurassic as the starting point of our analysis is motivated by geodynamic considerations on the mantle transit time, to remain within a timescale comparable to a mantle overturn [33]. We then analyse spreading rate variations and hiatus surfaces, finding they frequently correlate, except when plate boundary forces may play a significant role. Section 5 places our results into the context of geological studies. This is followed by a discussion on geodynamic implications, where we employ Poiseuille/Couette models for scale analysis. Finally, we draw conclusions in §6.

## 2. Oceanic spreading rates

**Figure 2.**
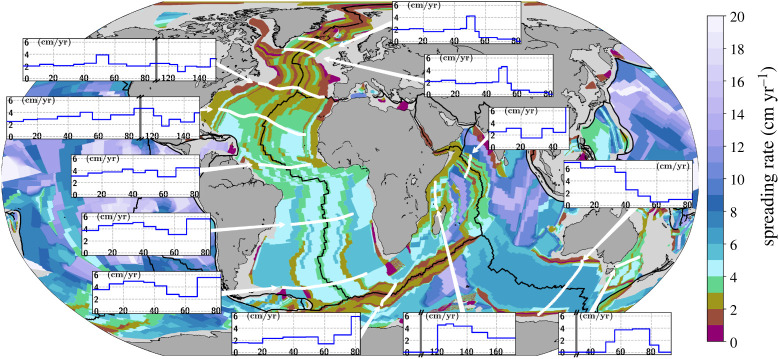
Grid of ocean floor spreading rates reproduced from Seton *et al.* [50] based on GTS2012 [51] timescale. Colours indicate spreading rates at time of crustal creation, revealing frequent spreading rate changes throughout the oceans. Inset figures, where y-axis is spreading rate in $\mathrm{cm}\;{\mathrm{yr}}^{-1}$ and x-axis time in Ma, show rates at selected locations. Grey vertical lines in the inset plots for the Central Atlantic correspond to the Cretaceous Normal Superchron (CNS), when spreading rates are known only at mean rates across the CNS (see text). For the Indian ocean, they represent omitted time. Profiles extracted from [52] database using *pyGPlates* [53]. (Online version in colour.)

Figure 2 presents the spreading rates of the seafloor at the time of crustal creation as a data grid from Seton *et al.* [50] based on the GTS2012 [51] timescale (see [54] for a discussion of the influence of the timescale choices for the uncertainty of reconstructed spreading histories). We also superpose profiles of relative spreading rates throughout the Atlantic, the Indian and the Southern Ocean south of Australia, extracted with *pyGPlates* [53] from the latest dataset of Müller *et al.* [52], to bring out local spreading rate variations. Most of the inset spreading rate profiles are reported for the time from 80 Ma onward, whereas profiles in the oldest ocean floor regions, such as the Central Atlantic and Madagascar, are shown from earlier times onward. The Cretaceous Normal Superchron (CNS) restricts the temporal resolution of Cretaceous spreading rates to mean values [27,55], therefore we omit the report of spreading rates spanning this time period.

Overall, figure 2 reveals that rapid spreading rate variations (less than 10 Myrs) occurred throughout the oceans, with spreading rates in the South Atlantic and the Antarctica-Australia spreading centre ($∼6\;\mathrm{cm}\;{\mathrm{yr}}^{-1}$) being faster compared to rates in the North Atlantic ($∼2\;\mathrm{cm}\;{\mathrm{yr}}^{-1}$). The inset profiles from north to south for the Atlantic show distinct temporal variations. The North Atlantic rates have a noticeable peak of $∼4\;\mathrm{cm}\;{\mathrm{yr}}^{-1}$ in the early Eocene from ∼55–45 Ma that follows the onset of spreading in the region. They drop to $∼2\;\mathrm{cm}\;{\mathrm{yr}}^{-1}$ in the last 45 Ma. The South Atlantic is characterized by higher rates and larger variations. At 80 Ma, near the end of the CNS, nominal rates are $∼6\;\mathrm{cm}\;{\mathrm{yr}}^{-1}$. In the Paleocene, from ∼65 to 55 Ma, they drop by a factor of three to $∼2\;\mathrm{cm}\;{\mathrm{yr}}^{-1}$, followed by a renewed increase up to a peak of $∼6\;\mathrm{cm}\;{\mathrm{yr}}^{-1}$ at ∼30 Ma. From ∼25 Ma onward, rates decrease to $∼4\;\mathrm{cm}\;{\mathrm{yr}}^{-1}$. The Central Atlantic is a superposition of trends from the south, i.e. slow rates in the Paleocene ($∼3\;\mathrm{cm}\;{\mathrm{yr}}^{-1}$) around 60 Ma, and from the north, i.e. a peak in the early Eocene at ∼50 Ma ($∼4\;\mathrm{cm}\;{\mathrm{yr}}^{-1}$). The spreading rate profile between India and Africa in the Indian ocean shows two peaks of $∼3\;\mathrm{cm}\;{\mathrm{yr}}^{-1}$ each. One lasts from ∼40 to 35 Ma, the other from ∼20 to 10 Ma, separated by a minimum of $∼1.5\;\mathrm{cm}\;{\mathrm{yr}}^{-1}$ from ∼35 to 20 Ma. Prior to 45 Ma, spreading velocities are significantly higher ($∼14\;\mathrm{cm}\;{\mathrm{yr}}^{-1}$). The Lord Howe Rise profile (east of Australia) shows high spreading rates ($∼4\;\mathrm{cm}\;{\mathrm{yr}}^{-1}$) from ∼80–60 Ma decreasing to zero by ∼50 Ma. Conversely, spreading rates in the Southern Ocean south of Australia are near zero ($∼0.5\;\mathrm{cm}\;{\mathrm{yr}}^{-1}$) from 80 to 60 Ma, increasing from the Paleocene onwards to spreading rates of $∼6\;\mathrm{cm}\;{\mathrm{yr}}^{-1}$.

## 3. Continental Base Hiatus Surfaces

**Figure 3.**
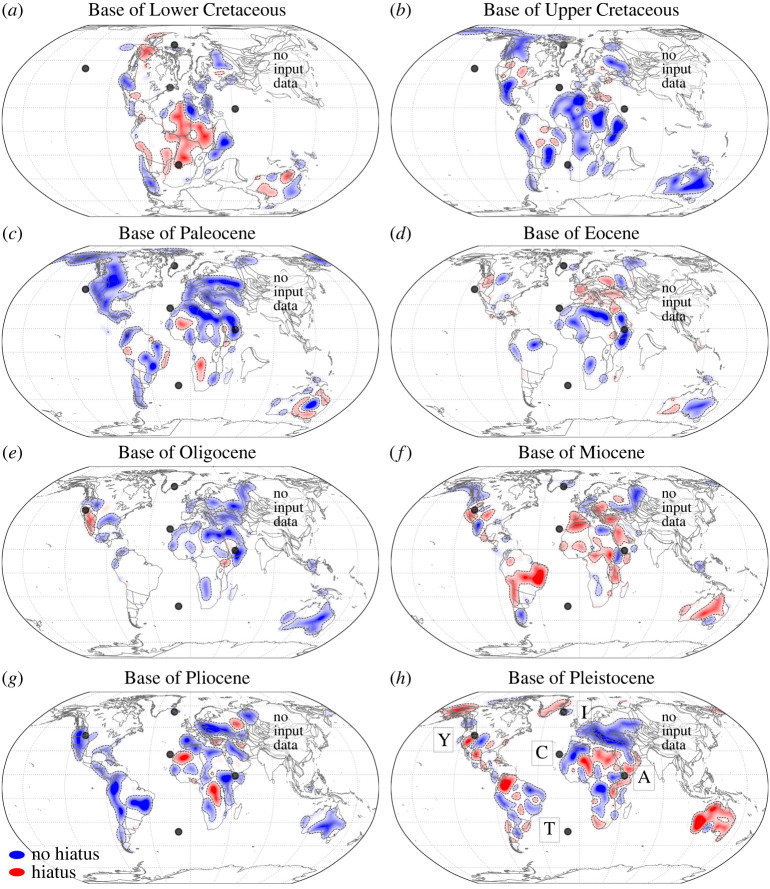
Base Hiatus Surfaces (BHS) from Hayek *et al.* [45,46] for eight geological series from Base of Lower Cretaceous to Base of Pleistocene (*a*–*h*) reconstructed paleogeographically with a global Mesozoic–Cenozoic plate motion model [27] tied to a reference frame of global moving hotspot and a True Polar Wander (TPW) corrected paleomagnetic reconstruction [56], with the latter updated by Seton *et al.* [55] (see text), and placed into a plate tectonic configuration corresponding to the base of each geological series. Red/blue colours represent the hiatus/no hiatus surfaces. Black dotted lines contour the spherical harmonics signal at the ±0.1 amplitude. Each map serves as a proxy for paleotopography (red=high, blue=low) in the preceding series (see text). Black dots are current locations of Yellowstone (Y), Canaries (C), Afar (A), Iceland (I), Tristan (T) hotspot [57]. (Online version in colour.)

Figure 3 shows Base Hiatus Surfaces (BHS) taken from Hayek *et al.* [45,46] for North and South America, Europe, Africa and Australia for eight geologic series beginning with the Lower Cretaceous. The resolution of geological series is chosen, because this is the most frequently adopted temporal resolution for interregional geologic maps [42]. The choice of the Lower Cretaceous as the oldest stratigraphic unit represents the mantle transit time [33], as noted before. We use the terms *un/conformable* and *hiatus/no hiatus* indistinguishably and follow Friedrich [42], Carena *et al.* [44] and Hayek *et al.* [45]. They define *hiatus/unconformable* as the state when one or more geological series in the chronostratigraphic chart [47,48] immediately preceding the target geological series are missing, regardless of whether either geological series has missing stages. *No hiatus/conformable* is defined as the complementary state. Hiatus is delimited for a given target geological series by any occurrence of the immediately preceding geological series. From hiatus mapped this way, Hayek *et al.* [45,46] construct BHS through a spherical harmonic representation (up to degree 15 and convolved with a Gaussian filter), reconstructed to their past tectonic setting with a global Mesozoic–Cenozoic plate motion model [27] tied to a global moving hotspot reference frame [56] from present-day to 100 Ma and a True Polar Wander (TPW) corrected paleomagnetic reconstruction [58] for times older than 100 Ma. The latter includes a longitudinal shift of $10^{\circ }$ incorporated by Seton *et al.* [55]. The surfaces are presented in the plate configuration that corresponds to the base of each geological series.

BHS serves as a proxy for paleotopography and vertical motion of the continents, as suggested by Friedrich *et al.* [41] and earlier authors (e.g. [59]). Red/blue colours depict un/conformable (hiatus/no hiatus) contacts, respectively, indicative of high/low topography in the preceding geological series. Blank regions reveal the absence of the target geological series and its immediately preceding unit. Such regions may have undergone intense and/or long-lasting erosion or non-deposition, indicative of intense and/or persistent exhumation and surface uplift [41–46]. Black dots in each BHS figure mark the current location of the Yellowstone, Canaries, Afar, Iceland and Tristan hotspots [57]. A detailed presentation of the data sources, the BHS construction and the related uncertainties are given in Hayek *et al.* [45].

**Figure 4.**
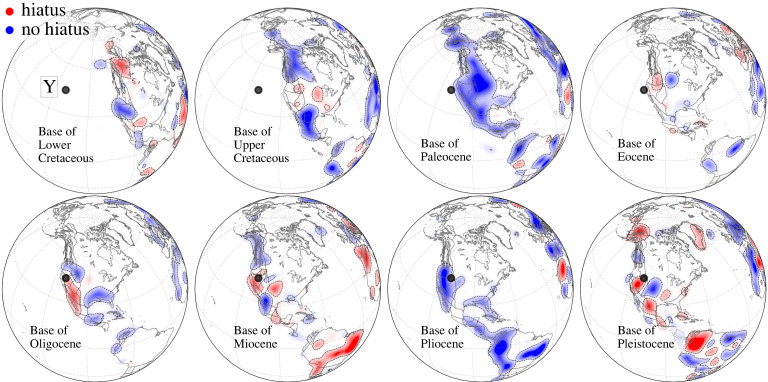
Same as figure 3, but centred on North America. Each BHS map serves as a proxy for paleotopography (red=high, blue=low) in the preceding geological series (see text). Black dot is current Yellowstone (Y) hotspot location [57]. From the Base of Eocene to Base of Miocene hiatus signal surrounds the current location of Yellowstone. (Online version in colour.)

Overall figure 3 reveals significant differences in hiatus distribution across and between continents at the timescale of geologic series. Described in detail in Hayek *et al.* [45,46], we summarize the main BHS features and recall that BHS at each geological series serves as a proxy for topography in the preceding geological series. *Base of Lower Cretaceous*, figure 3*a*, shows hiatus surface (red) and blank regions, indicative of high topography in the Upper Jurassic, in much of North and South America, Africa and Australia. No hiatus (blue), indicative of low Upper Jurassic topography, is prominent in northernmost Africa and Europe. *Base of Upper Cretaceous*, figure 3*b*, shows hiatus and blank regions in parts of Europe, North and South America. No hiatus is prominent throughout much of Africa, Australia and South America in the Paraná region. *Base of Paleocene*, figure 3*c*, reveals isolated patches of hiatus surface and blank regions along the east coast of Brazil, in Southern Africa (Karoo Basin) and Australia. No hiatus regions dominate elsewhere throughout the continents. *Base of Eocene*, figure 3*d*, presents hiatus surface and blank regions prominently in two continents: Europe, and South America except the Amazon Basin. No hiatus exists in the northernmost part of Africa, the Karoo Basin and eastern Australia. *Base of Oligocene*, figure 3*e*, displays blank regions in Africa. But the foremost occurrence is in South America, where it signals an almost complete absence of Oligocene and Eocene strata throughout the continent. Limited hiatus surface exists in the western part of North America and the Afar region. Europe, northernmost Africa, the Karoo Basin and much of Australia show prominent no hiatus regions. In *Base of Miocene*, figure 3*f*, hiatus surfaces dominate across the continents. Prominent examples include North and South America, parts of Europe, Australia and Africa. North America shows conformable contacts surrounding the hiatus regions near the Yellowstone hotspot location. Alaska, Patagonia and Central Europe also show conformable contacts. *Base of Pliocene*, figure 3*g*, reveals minor hiatus surface and blank regions. Hiatus is located in central Africa and near the Canaries. Blank regions occur in eastern North and South America. No hiatus signals dominate elsewhere. *Base of Pleistocene*, figure 3*h*, shows North and South America and Africa with a mix of hiatus and no hiatus surfaces. Extensive hiatus surface exists in Australia, except the *Nullarbor Plain*, Alaska, the eastern margin of Greenland and north-central Africa. No hiatus surface prevails in Europe and the Congo Basin. Figure 4 shows BHS with a view centred on North America, to illuminate a continent moving towards a hotspot. Much of the eastern half of the continent reveals blank regions, reflecting the absence of Lower Cretaceous to Pleistocene series. But, in the western half of the continent, the hiatus surface changes at interregional scales while North America approaches the current location of the Yellowstone hotspot. A prominent change occurs from the *Base of Paleocene* to *Base of Eocene*, when an extensive no hiatus signal transforms to mostly blank regions and hiatus, indicative of growing topography. From the *Base of Eocene* to *Base of Miocene* hiatus signal surrounds the current location of Yellowstone, starting with hiatus to the northeast of the current plume location and leading to hiatus located in the Columbia Plateau regions and the Interior Plains, as noted also by Friedrich *et al.* [41] and Stotz *et al.* [60].

## 4. Oceanic spreading rates and continental Base Hiatus Surfaces

### (i) Atlantic Realm

**Figure 5.**
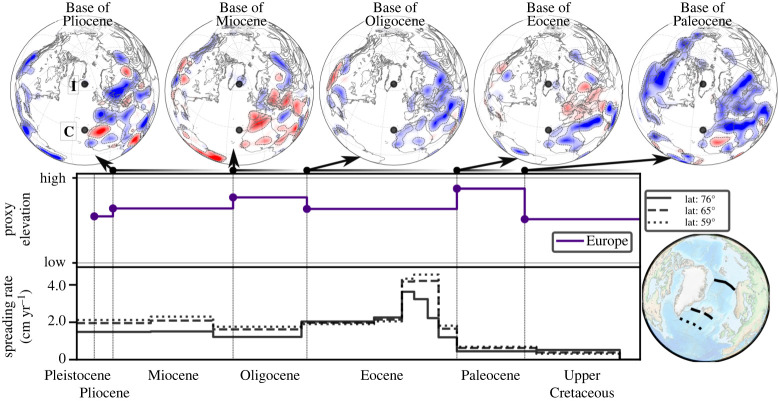
Top row shows BHS centred on the North Atlantic from Base of Pleistocene to Base of Paleocene (see figure 3 caption), with black dots showing the current hotspot locations of Iceland (I) and Canaries (C) [57]. Middle row shows proxy elevation obtained by the sum of hiatus surfaces and blank regions (see text) for a given geological series normalized by the total area under consideration (Europe). Bottom row shows spreading rates for three transects as indicated in bottom right globe. X-axis shows the time range from Pleistocene to Mid Upper Cretaceous. The BHS show prominent hiatus surfaces at the Base of Eocene and Base of Miocene, reflected also as high proxy elevation for the Paleocene and Oligocene. The two proxy elevation peaks are followed by two periods of elevated spreading velocity in the early Eocene and early Miocene. (Online version in colour.)

**Figure 6.**
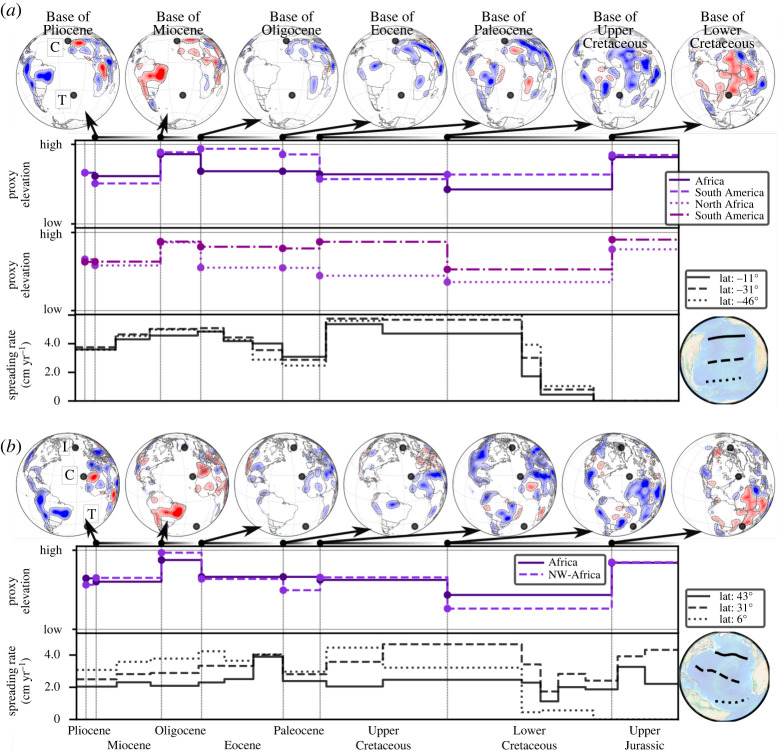
(*a*) Same as figure 5 with top row showing BHS with a view centred on the South Atlantic from Base of Pleistocene to Base of Upper Cretaceous. Black dots represent current hotspot locations of Canaries (C) and Tristan (T) [57]. Middle rows show proxy elevation curves (see text) for Africa and South America (top) and northern and southern Africa (bottom) from Pleistocene to Lower Cretaceous (see text). Bottom row shows spreading rates for three transects as indicated in bottom right globe. Elevated Jurassic proxy elevation for Africa and South America precedes the spreading onset in the Lower Cretaceous. An increase in Upper Cretaceous African proxy elevation owing to growing elevation in the southern part of the continent (see southern Africa proxy elevation curve) goes along with high Cretaceous spreading rates (see text). A Paleocene increase in South American proxy elevation precedes elevated Eocene spreading rates (see text). (*b*) Same as (*a*) for the Central Atlantic. Black dots in the BHS represent current hotspot locations of Iceland (I), Canaries (C) and Tristan (T) [57]. Middle row shows proxy elevation curves for Africa and its northwestern portion (see text), while bottom row shows three spreading rate transects as indicated by bottom right globe. The northern/southern spreading rate profiles resemble the history of the North/South Altantic respectively (see text). (Online version in colour.)

Figure 5 (top row) shows BHS with a view focused on the North Atlantic/Europe. There are two periods of widespread hiatus in Europe, in the Paleocene and the Oligocene, revealed by hiatus surface at the *Base of Eocene* and *Base of Miocene*, respectively. This is illustrated by a curve (middle row) for proxy elevation, which we construct as the sum (for a given geological series) of hiatus and blank regions, normalized by the total area (Europe) under consideration. The bottom row plots oceanic spreading rates at three transects, north to south, between Greenland and Europe from the beginning of the Upper Cretaceous onward. The spreading rate magnitude increases, as expected, with decreasing latitude and further distance of the transects from the Euler Pole. Importantly, the transects reveal two periods of higher spreading rates: a 10 Myrs peak in the early Eocene and a second increase in the early Miocene. The latter is more pronounced in the two southern profiles. These observations were noted by Vibe *et al.* [43]. Figure 6*a* (top row) displays BHS with a view focused on the South Atlantic. Two periods of widespread hiatus exist for much of Africa and South America, in the Upper Jurassic and the Oligocene, revealed by hiatus surface at the *Base of Lower Cretaceous* and *Base of Miocene*. There are also two periods of prominent blank region (absence of the considered geological series and its immediately preceding unit) for both continents at the *Base of Eocene* and *Base of Oligocene*. That means the maps show neither Paleocene, nor Eocene and Oligocene strata in much of Africa and South America. In the southern half of Africa blank regions also dominate the *Base of Paleocene*. The proxy elevation curves for Africa and eastern South America, figure 6*a* (top part of the two middle rows) normalized to the area of Africa and eastern South America, respectively, bring this out. They show elevated Jurassic topography for both continents, followed by lower topography in the Cretaceous. The African curve increases in the Upper Cretaceous, due to growing proxy elevation in the southern part of the continent, followed by a pronounced continent-scale Oligocene increase. The South American curve instead increases markedly in the Paleocene, and reaches an Eocene peak value. Africa is a large continent, and likely responds to different dynamic topography domains due to its size (see Hayek *et al.* [45,46] and Carena *et al.* [44] for a discussion). To this end, we bring out the different topographic evolution of northern and southern Africa by two proxy elevation curves (lower part of the two middle rows) that are constructed for the northern (plate IDs 714, 715, 503 from the Matthews *et al.* [61] dataset) and southern portions (plate IDs 701, 709, 712, 713 [61]) of the continent, respectively. The curves show a noticeable increase of Upper Cretaceous proxy elevation for southern Africa. Figure 6*a* (bottom row) reports three spreading rate transects for the South Atlantic. Spreading rates are elevated for much of the Cretaceous and the Eocene to mid-Miocene, separated by lower spreading rates in the Paleocene. These observations were noted by Colli *et al.* [32]. Figure 6*b* (top row) depicts BHS with a view centred on the Central Atlantic and northwest Africa. The maps show two hiatus periods, in the Jurassic and the Oligocene, indicated by hiatus surface at the *Base of Lower Cretaceous* and *Base of Miocene*. Two proxy elevation curves, constructed for all of Africa or only for its northwestern sub-region (plate ID 714 [61]), are plotted in figure 6*b* (middle row). Both curves are similar. But the lower value for proxy elevation in the Lower Cretaceous and the peak in proxy elevation for the Oligocene is more pronounced in the northwestern sub-region. Figure 6*b* (bottom row) displays spreading rates for three Central Atlantic transects. They differ from each other in that the northernmost/southernmost transect resembles the spreading rate history of the North/South Atlantic, respectively. For instance, a spreading rate increase in the Lower Cretaceous relates to the South Atlantic transect, while a peak in the early Eocene resembles the spreading rates of the North Atlantic.

### (ii) Indo-Australian realm

**Figure 7.**
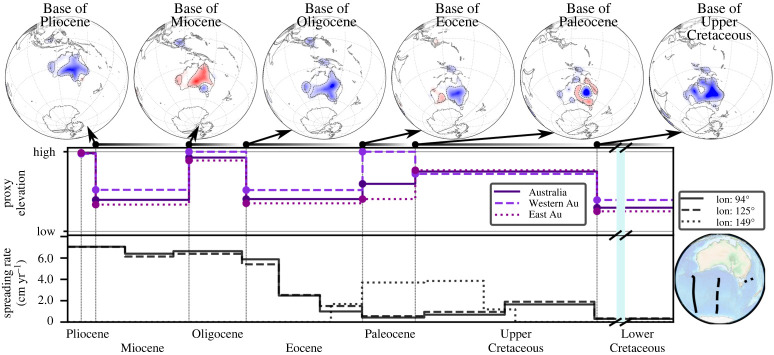
Same as figure 5 with view centred on Australia. Top row shows BHS (see text). Middle row shows proxy elevation curve for the entire continent or separated for eastern and western Australia (see text). Bottom row shows spreading rates for three transects as indicated in bottom right globe. Blue vertical line in the Lower Cretaceous represents a time-lapse omission. Elevated Upper Cretaceous proxy elevation goes along with spreading of Lord Howe Rise (dotted curve), whereas elevated western Australia proxy elevation in the Paleocene precedes rapid Eocene spreading between Antarctica and Australia (see text). (Online version in colour.)

**Figure 8.**
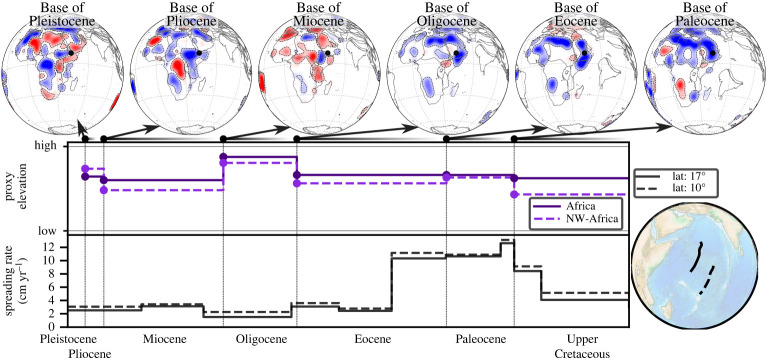
Same as figure 5 with view centred on Eastern Africa. Top row shows BHS (see text) with black dot representing the current hotspot location of Afar (A) [57]. Middle row shows proxy elevation curve for the entire continent or its northeast subregion (see text). Bottom row shows spreading rates for two transects as indicated in bottom right globe. Growing East Africa proxy elevation in Paleocene–Oligocene and decreasing Carlsberg spreading rates, presumably reflect plate boundary forces from India-Asia Collision (see text). (Online version in colour.)

Figure 7 (top row) shows BHS with a view focused on Australia. Extensive conformable area exists at the *Base of Upper Cretaceous*, indicative of low topography in the Lower Cretaceous. Hiatus and blank regions, apart from the Eromanga basin, dominate the *Base of Paleocene*, indicative of overall higher Upper Cretaceous topography. A distinct difference between western and eastern Australia emerges at the *Base of Eocene*, when hiatus/no hiatus surface dominates the western/eastern portion of the continent, respectively. The difference continues at the *Base of Oligocene*, with a blank surface in much of northwestern Australia and no hiatus elsewhere. There is a near continent-wide hiatus surface at the *Base of Miocene*, followed by extensive no hiatus at the *Base of Pliocene*. Proxy elevation curves, figure 7 (middle row), for the entire continent or separated for eastern and western Australia (the separation corresponds to the Western Australia state border, west of $129^{\circ }$ present-day longitude) bring these observations out. The continent as a whole experiences an overall proxy elevation increase from the Lower to the Upper Cretaceous and an Oligocene peak value. But there is a marked difference for the eastern and western Australia proxy elevation curves in the Paleocene. The former decreases, the latter increases relative to the Upper Cretaceous. We plot spreading rate transects for the Antarctic-Australia ridge and the Lord Howe Rise in the bottom row of figure 7. The Lord Howe Rise was active to the southeast of Australia from the middle of the Upper Cretaceous onwards, with velocities of $∼4\;\mathrm{cm}\;{\mathrm{yr}}^{-1}$. It ceased spreading in the early Eocene, when motion initiated along the Antarctica-Australia ridge. From then on Antarctica-Australia spreading rates increased significantly, reaching $∼6\;\mathrm{cm}\;{\mathrm{yr}}^{-1}$ by the Oligocene. The top row of figure 8 displays BHS with a view centred on East Africa, from the *Base of Oligocene* onward. This links to the rise of the Afar plume, for which domal uplift, indicated as well in our maps, has been documented after the early Eocene [62]. Even though the main hiatus surface occurs at the *Base of Miocene*, there is another hiatus at the *Base of Pleistocene*. Proxy elevation curves, figure 8 (middle row), for all of Africa or its northeast sub-region (plate IDs 709, 710, 712, 713, 715, 503 from the Matthews *et al.* [61] dataset) bring this out. The Pliocene increase is more pronounced in the regional proxy elevation curve of East Africa compared to the curve for all of Africa. Figure 8 (bottom row) plots two spreading rate transects for the Carlsberg Ridge since the Upper Cretaceous. They show minor spreading rate variations from the mid-Eocene onwards.

## 5. Discussion

It is a geodynamic tenet that plates organize the flow and that they are an integral part of the convective system (see [63] for a review). Yet the precise nature of how plate motions are caused by mantle convection remains incomplete, because the strength of plates conceals the underlying flow. The description by Morgan *et al.* [16] and Höink *et al.* [19–22] of asthenosphere flux in terms of Poiseuille/Couette flow offers a way to overcome this difficulty by mapping upper mantle flow through its topographic and viscous effects. This geodynamic perspective motivates us to compare changes of oceanic spreading rates and continental hiatus surface, by building upon earlier work and exploiting growing observational constraints on both.

A link between spreading rate variations and dynamic topography is perhaps best evinced for the North Atlantic (figure 5), where two periods of expanding hiatus surface across Europe in the Paleocene and Oligocene precede the Eocene spreading onset and faster Miocene spreading rates, as noted by Vibe *et al.* [43]. The region is well suited for an observational geodynamic analysis. Its Cenozoic opening history has left well-preserved sea-floor magnetic lineations (e.g. [64,65]) and hiatus surfaces. There are also well-documented regional uplift episodes reported, for instance, for the British Isles, Greenland, Scandinavia and Central Europe (e.g. [66–69]). Reviews of uplift and subsidence events across the region are given by various authors [70–72]. The region also contains prominent plume systems. They provide a geodynamically plausible mechanism to drive active upper mantle flow, as suggested by scaling analysis [21]. Plume systems at the western edge of Europe include the Canaries, which experienced Oligo/Miocene uplift at the local [73] and regional scale [74], and the Iceland-Jan Mayen system. The broad extent of the latter was imaged seismically by Rickers *et al.* and Celli *et al.* [75,76]. Importantly, studies of the Iceland-Jan Mayen system reveal temporal mass flux [77] and thermal anomaly [78] variations, with peaks in the early and late Cenozoic. Our results support these accounts of temporal system variations and indicate their interregional scale, documenting early Paleogene and Neogene changes in dynamic topography and spreading rates, induced presumably through variations in pressure-driven upper mantle flow.

The opening history of the South Atlantic realm, which hosts the Tristan hotspot, spans nearly three times that of the North Atlantic, reaching back into the Mesozoic. But links between spreading rates and dynamic topography are more difficult to establish, because the early South Atlantic opening falls largely within the CNS, restricting the interpretation of Cretaceous spreading rates to mean values, and because the long duration of the Lower/Upper Cretaceous series (approx. 40 Myrs each) limits the temporal resolution of our geological series-based hiatus mapping during that time. The main observation is a bimodal spreading rate distribution, with Cretaceous and mid-Cenozoic peaks separated by a Paleocene low, as seen in our results and pointed out by Colli *et al.* [32]. This goes along with high Upper Jurassic proxy elevation for Africa and South America, and renewed proxy elevation for southern Africa in the Upper Cretaceous and for South America starting in the Paleocene. But observations beyond this first order division are beginning to emerge. For the phase preceding the South Atlantic opening, Krob *et al.* [79] use geological archives (stratigraphic and thermochronological data) and stratigraphic frameworks to document interregional-scale Upper Jurassic uplift in South America and Africa, which they link to the Paraná-Etendeka plume rise well in advance of flood basalt eruptions and the onset of seafloor spreading. For the CNS, and using a new identification of magnetic anomalies located within that time period, Granot & Dyment [80] report accelerating rather than constant South Atlantic spreading rates, with a peak achieved in the early Campanian (approx. 80 Ma). This goes along with increasing Upper Cretaceous proxy elevation in southern Africa (figure 6*a*) and a growing consensus about the uplift history of the South African Plateau (SAP). Reports on the latter assign Upper Cretaceous ages to peak sediment flux (e.g. [81–83]) around the SAP, increased kimberlite occurrence in southern Africa [84], and basin inversion and margin tilting along the Namibian coast [85,86], consistent with a Late Cretaceous SAP uplift pulse inferred from thermochronological studies [87,88]. No major coeval activity is indicated for the Tristan hotspot. But the ∼85 Ma eruption of the Marion hotspot [89,90] occurs at a geodynamically relevant distance (approx. 2000 km east of southern Africa) addressed in the next section. The Tertiary phase of the South Atlantic opening has accelerated spreading rates starting in the Eocene, preceded by growing South American proxy elevation (figure 6*a*) in the Paleocene. The interregional scale of this event is evinced by a variety of studies. They document a Paleogene hiatus in Andean Foreland Basins [91], regional uplift along the Argentine margin [92], and an Eocene reactivation of the South American passive margin (e.g. [93–95]) deduced from landscape analysis and thermochronological data. Far field effects from plate boundary forces associated with the Andean margin [93] have been invoked to explain these events. But most studies agree that prominent uplift of the Andes started in the mid Eocene, reaching a peak in the Oligocene, with a second uplift period in Late Miocene [96]. It is not obvious how increased topographic loads from the Andes would induce faster South Atlantic spreading. Instead, there are reports for anomalously young (late-stage) volcanism (≈46 Ma) on the Rio Grande Rise (RGR) and an Eocene subaerial exposure of the RGR at Drill Site 516 [97,98]. They point to temporal flux variations of the Tristan hotspot, similar to what is reported for Iceland. This should be considered in future geodynamic analyses of the basin. A review of Cretaceous-Cenozoic sediment supply to the South Atlantic margins and the complex uplift and subsidence history of the region is given by MacGregor [99].

Links between spreading rates and dynamic topography for Australia must acknowledge the onset of subduction in the Cenozoic of the Indo-Australian plate beneath Southeast Asia (e.g. [100]), so that the geodynamic setting of this region differs fundamentally from the Atlantic realm. Australia’s dynamic topography history from the Jurassic onward has been reviewed by Harrington *et al.* [101] and involves several key observations. Long-wavelength tilting since the late Cretaceous (e.g. [102,103]) occurred when the continent approached the subduction systems of Southeast Asia on its northward passage. It is manifested by high Miocene subsidence rates on the Australian northwest shelf inferred from the stratigraphic architecture of carbonate platforms [104]. River profile studies provide additional constraints. They report regional uplift for western and central Australia starting in the Eocene (e.g. [105,106]) and a two-stage uplift history for the Eastern Highlands. A first stage, from 120 to 80 Ma, coincided with rifting along the eastern margin, whereas a second stage, inferred broadly for 80–10 Ma, formed the Great Escarpment [106]. A third and long-standing observation relates to Australia’s flooding record. Its maximum occurs in the Lower Cretaceous, when the Eromanga and Sutra basins in the eastern half of the continent experienced marine inundation. The discrepancy of Australia’s flooding record with global sea-evel curves (e.g. [107]) was noticed early on and prompted pioneering geodynamic studies that attributed the inundation to Australia’s passage over a slab associated with Gondwana subduction (e.g. [108,109]). Our results agree with these findings and document the interregional nature of Australia’s topographic changes. They show that spreading onset of the Lord Howe Rise went along with a proxy elevation growth from the Lower to the Upper Cretaceous, although a detailed analysis is limited by the long duration of the Lower/Upper Cretaceous series. The interregional scale of this elevation change suggests the involvement of active mantle flow. Several studies have attributed Lord Howe spreading to a mantle upwelling (e.g. [110–112]). Supporting evidence comes from geochemical data tracing a deep mantle (HIMU) component in Upper Cretaceous volcanics in Zealandia [113]. Active plume-driven flow in the Upper Cretaceous at Australia’s eastern edge would indeed provide a plausible link between the topographic and spreading signal at that time. By contrast, no plume activity has been invoked, to our knowledge, for Western Australia’s proxy elevation growth in the Paleocene, which preceded the Eocene onset of Antarctic/Australia separation (figure 7). However, Stotz *et al.* [114] used coupled mantle circulation and tectonic models to suggest that Poiseuille flow inherited from Mesozoic mantle circulation led to the Eocene separation of Australia and Antarctica, eventually inducing subduction of the Indo-Australian plate beneath Southeast Asia and high spreading rates along the Antarctica-Australia ridge.

**Figure 9.**
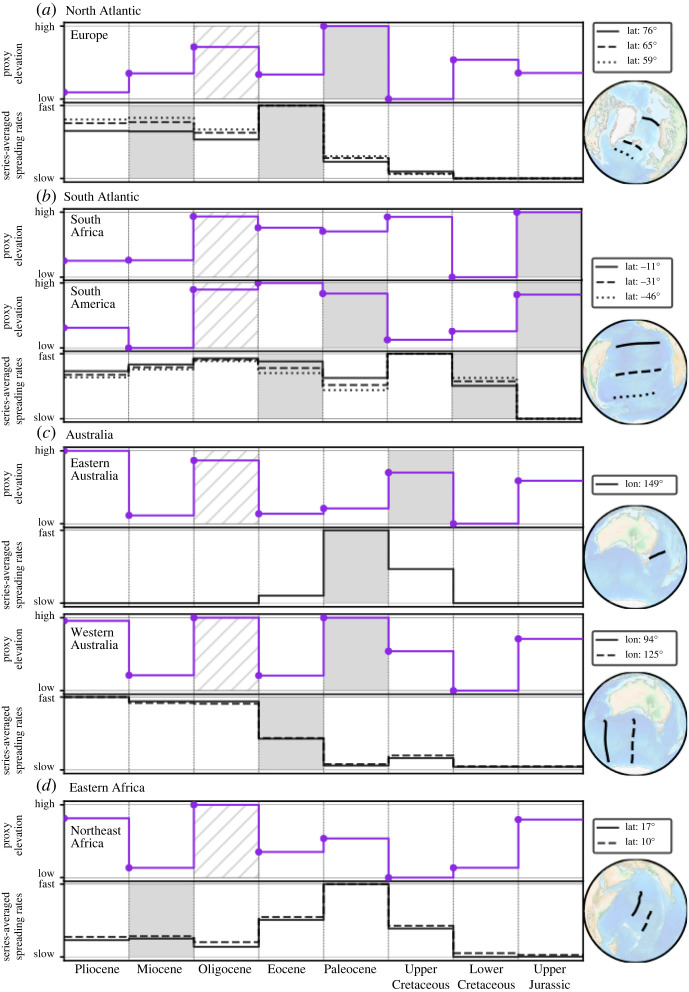
Summary figure showing correlation between high proxy elevation (PE) and subsequent spreading rate changes with grey boxes for studied regions from Upper Jurassic to Pliocene. PE curves (dot marks the datum) are normalized to their minimum/maximum, spreading rates are averaged over each geological series, and all series are represented with the same length. (*a*) North Atlantic: high Paleocene PE followed by Eocene spreading onset. (*b*) South Atlantic: high Upper Jurassic South African/South American PE followed by Lower Cretaceous spreading onset. High Paleocene South American PE followed by Eocene spreading rate increase. (*c*) Australia: high Upper Cretaceous Eastern Australia PE followed by peak Paleocene Lord Howe Rise spreading rates. High Western Australia Paleocene PE followed by Eocene Antarctica-Australia spreading onset. (*d*) Eastern Africa: growing Paleo/Oligocene PE and simultaneous decreasing Carlsberg spreading rates presumably reflect boundary forces from India-Asia collision. High Oligocene PE (hatched box) in part reflects eustatic signal from Antarctic glaciation onset [45,46]. Long duration of Upper/Lower Cretaceous series combined with Cretaceous Normal Superchron mask relation between PE and spreading rate changes (e.g. high Upper Cretaceous South African PE and South Atlantic spreading rates, see text).

Links between dynamic topography and plate motion variations are perhaps least evinced in our maps for the Indian Ocean realm, because collisional forces associated with India-Eurasia convergence arguably make this ocean basin the one currently most strongly influenced by plate boundary forces. Several studies argue for significant edge forces in the region as a result of topographic loads associated with the region's orogenic plateaus [115,116], amounting to $∼5\text{--}10\times 10^{12}\;N\;m^{-1}$ [117,118], sufficient to reduce India’s plate motion [117]. Additional complexity seemingly arises from temporal flux variations of the Reunion plume [119,120]. Not surprisingly, our results reveal growing East Africa proxy elevation in the Paleocene–Oligocene and simultaneous decreasing Carlsberg spreading rates, which presumably reflect expanding boundary forces from India-Asia collision, and minor spreading rate variations in the late Paleogene and Neogene, at the time of the Afar plume arrival. Figure 9 summarizes our findings and reveals the correlation between high proxy elevation and subsequent spreading rate changes with grey boxes for the studied regions from Upper Jurassic to Pliocene.

Our discussion must acknowledge the influence of eustatic contributions to the proxy elevation curves. For instance, there is an increase in the Oligocene proxy elevation curve for all regions considered, see hatched area in figure 9. In Hayek *et al.* [45,46] we bring out the sea-level signal by plotting the temporal evolution of no-/hiatus surfaces in the BHS separately for individual continents and combined for all continents considered. This reveals two prominent continent-wide maxima in the BHS at the Base of Miocene and Base of Pleistocene corresponding to high proxy elevation curves in the Oligocene and the Pliocene, respectively. The latter coincide with the onset of glaciation in Antarctica and the Northern Hemisphere, respectively. This suggests caution in the interpretation of Oligocene and Pliocene hiatus.

Our discussion of the comparison of hiatus surfaces and past plate motion changes must emphasize the severe limitations of the input data for our hiatus mapping method. Addressed in detail by Hayek *et al.* [45], we recall first that the BHS are well constrained in lateral extent but not in amplitude. The latter requires independent calibration, for example, by using thermochronological data [121]. Second, that the temporal resolution of interregional hiatus analysis depends on the temporal resolution of the input geological maps. At continent scale, they are currently limited to the geological series level. The limitation is aggravated because hiatus is likely longer than indicated by the missing geological series (see fig. 3 in [42]). At any one location sedimentary successions may represent only a small portion of a geological series. This implies large temporal uncertainties in our analysis, inevitably hiding shorter duration lacunae and favouring large time intervals. The severe limitations of the input data can be alleviated with additional geologic indicators, which are beginning to yield powerful constraints on past dynamic topography. They include paleoaltimetry estimates [122], studies of river profiles (e.g. [123]), landforms [124] and sediment provenance [62,125], constraints from thermochronological [126–128] or paleobiological and paleoenvironmental data [129], as well as quantifications of sediment budgets at the scale of continental margins [81–83,130]. Observational constraints on current and past dynamic topography are reviewed very effectively by Hoggard *et al.* [131]. Efforts to better constrain past continental vertical motion are matched by similar efforts to improve our knowledge of horizontal past plate motion. Progress is underway to map past plate velocities from sea-floor magnetic lineations at temporal resolutions of ∼1 Myrs or less (e.g. [28,29]) when mitigating for finite-rotation noise [30]. In combination, these efforts should help to further constrain the recent geologic history of large-scale horizontal and vertical lithosphere motion, greatly assisting in geodynamic interpretations of plate driving and resisting forces.

### Geodynamic implications

Geodynamicists have long understood the effects of an asthenosphere on mantle flow in modulating the amplitude of dynamic topography and the horizontal flow range (e.g. [4,11]). Our analysis extends these results to geologic observations and suggests a timescale, on the order of a geological series, between the occurrence of continent-scale hiatus and plate motion changes. A similar timescale comes from fluid dynamic studies reporting a delay between domal uplift and magmatism above starting plumes (e.g. [132,133]). These studies are reviewed very effectively by Campbell [134]. The timescale is interpreted best through dynamic topography response functions of dynamic Earth models, because a weak upper mantle delays significant surface deflections into the final phase of material upwellings, when buoyant flow enters from the lower into the upper mantle (see [49] for a review). Our analysis also suggests a spatial scale for interregional hiatus, on the order of 2000–3000 km in diameter. The latter again agrees with starting plume studies, where it is attributed to plume heads flattening by lateral upper mantle flow [134].

**Figure 10.**
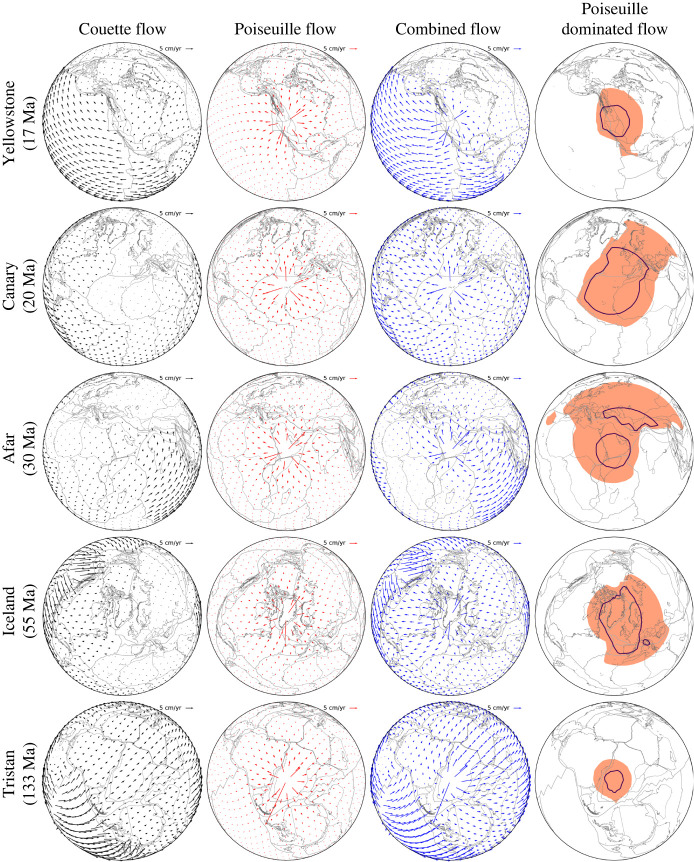
Upper mantle flow estimates based on Couette (black, first column) and Poiseuille (red, second column) models (compare figure 1), and the superposition of both (blue, third column), for a global Mesozoic–Cenozoic plate motion model [27] tied to a reference frame of global moving hotspot and TPW corrected paleomagnetic reconstruction [56], with the latter corrected by Seton *et al.* [55] (see text), shown at mid-asthenospheric level for the time when the Yellowstone, Canary, Afar, Iceland and Tristan plumes presumably arrived in the asthenosphere (see text for details). Poiseuille-dominated flow regions, as indicated by areas where Poiseuille flow exceeds Couette flow by at least $0.5\;\mathrm{cm}\;{\mathrm{yr}}^{-1}$, are shown in colour in column four. These regions are larger under slow-moving plates and comparable to the hiatus area inferred from our maps (compare figure 3, see text for details). Note the small size for the Poiseuille flow dominated region of Tristan compared to the other hotspots. Blue continuous line contours Poiseuille dominated area when the Poiseuille flow strength is reduced to half the reference value (see text). (Online version in colour.)

Our maps offer the opportunity to compare hiatus size with predictions from fluid dynamic models of Poiseuille/Couette flow (figure 1), following Stotz *et al.* [135]. Figure 10 shows analytic upper mantle flow estimates, derived from the assumption of Couette flow, Poiseuille flow and the superposition of both, at the time when the Yellowstone, Canary, Afar, Iceland and Tristan plumes presumably arrived in the asthenosphere, as evinced by the onset of widespread volcanism [57]. Couette flow (first column figure 10), induced in the underlying asthenosphere by tectonic plate motion, is computed from the reconstructions of Müller *et al.* [27] tied to a global moving hotspot reference frame [56] from present-day to 100 Ma and a TPW corrected paleomagnetic reconstruction [58] for times older than 100 Ma, with the latter including a longitudinal shift of $10^{\circ }$ incorporated by Seton *et al.* [55]. This flow is half the surface velocity at mid-asthenosphere depth. Poiseuille flow, induced by a plume-generated pressure gradient in the asthenosphere, is obtained from the equation

5.1 $$V_{\text{Poiseuille}}\approx \frac{D^{2}}{8\mu }\frac{\Delta p}{\Delta x},$$

where D is the asthenosphere thickness, and μ is its viscosity. Both values are tied together by inferences from post-glacial rebound (e.g. [136]). We choose a thickness of 110 km and a viscosity of $5\times 10^{19}\;\text{Pa}\;\text{s}$. Δp/Δx is the flow inducing pressure gradient, which we estimate from values for density contrast, gravity and dynamic topographic height in Δp=ρgh, respectively. We use a density contrast of $3300\;\mathrm{kg}\;m^{-3}$ and a height of 1400 m (e.g. [131]). Δx is the distance away from the plume centre. Equation (5.1) is singular at the plume centre Δx=0. To this end, we introduced a cut off at $15\;\mathrm{cm}\;{\mathrm{yr}}^{-1}$ which corresponds to a distance of ∼200 km from the plume source. Poiseuille flow (second column figure 10) is radially symmetric, as expected, decays away from the respective plume centres with velocities $>15\;\mathrm{cm}\;{\mathrm{yr}}^{-1}$, maintains velocities $∼5\;\mathrm{cm}\;{\mathrm{yr}}^{-1}$ at a distance of ∼1000 km from the plume, and decays further farther out. We note that our estimated Poiseuille flow velocities agree with inferences from geologic observations and dynamic models [137,138]. The combined flow (third column figure 10) is derived by adding up the Couette and Poiseuille flow as in Stotz *et al.* [135]. It varies geographically due to the Couette component, such that different locations experience different upper mantle flow for any given Poiseuille source. We highlight, as a proxy for active mantle flow, those areas where Poiseuille flow exceeds Couette flow by at least $0.5\;\mathrm{cm}\;{\mathrm{yr}}^{-1}$. These areas are larger under slow moving plates, as expected, and have an extent of $∼0.4\text{--}0.9\times 10^{7}\;{\text{km}}^{2}$, comparable to the hiatus area inferred from our maps. Our results allow us to evaluate, to first order, the ability of Poiseuille flow to initiate spreading rate changes, following Iaffaldano & Bunge [33].

5.2 $$A_{f}=\frac{\Delta v_{p}}{\Delta v_{f}}A_{p}.$$

Equation (5.2) relates the Poiseuille flow dominated area ($A_{f}$) to the area ($A_{p}$) affected by the spreading rate change. The latter is the entire plate. This relation is modulated by the ratio of the mean pressure-induced velocity change ($\Delta v_{f}$) to the plate velocity change, that is the spreading rate variation ($\Delta v_{p}$). It means that small Poiseuille flow dominated areas ($A_{f}$) can affect large plates areas ($A_{p}$), provided the mean pressure-induced velocity change ($\Delta v_{f}$) exceeds the plate velocity change ($\Delta v_{p}$). We assume a mean value $\Delta v_{f}$ $∼5\;\mathrm{cm}\;{\mathrm{yr}}^{-1}$ over a radius of ∼1500 km away from the plume centre, a Poiseuille flow dominated area $A_{f}$ $∼10^{7}\;{\mathrm{km}}^{2}$, and an average plate area $A_{p}$ $∼5\times 10^{7}\;{\mathrm{km}}^{2}$ for our analysis, noting that the current size of the African plate is $∼6.2\times 10^{7}\;{\mathrm{km}}^{2}$, whereas the size of the South American plate at the end of the Cretaceous, shortly before it resumed rapid spreading in the Eocene, was $∼2.5\times 10^{7}\;{\mathrm{km}}^{2}$. Our analysis shows that plume-driven flow may induce spreading rate changes ($\Delta v_{p}$) on the order of $∼1\;\mathrm{cm}\;{\mathrm{yr}}^{-1}$ for average-sized plates, comparable to the values reported in our study. Finally, we compute the linear force density associated with Poiseuille flow, for which we estimate plate basal shear stresses from the relationship

5.3 $$\tau =\mu \frac{\Delta v_{f}}{d},$$

where τ is the shear stress generated at the plate base, μ is the asthenosphere viscosity, $\Delta v_{f}$ is the velocity difference between plate and asthenosphere flow at mid depth and d is half the asthenosphere channel thickness. Taking an asthenosphere viscosity of $5\times 10^{19}\;\mathrm{Pa}\;s$, a velocity of $5\;\mathrm{cm}\;{\mathrm{yr}}^{-1}$, and a half-thickness of 55 km, as noted before, we obtain shear stresses of ∼1.5 MPa, in agreement with values reported from instantaneous and time-dependent geodynamic models [139–143]. The spatial coherence of Poiseuille flow evinced from our maps allows us to integrate the shear stress along flow lines. We take a distance of ∼1500 km, the radius achieved by Poiseuille flow dominated areas (fourth column figure 10) and obtain linear force densities of $∼2.25\times 10^{12}\;N\;m^{-1}$, comparable to ridge push values, which are estimated to $∼2\times 10^{12}\;N\;m^{-1}$ ([144] and references therein). Our estimates agree with earlier studies that have called for strong plume push forces [145], related, for instance, to the Reunion hotspot [119,146,147]. However, we point to the limitation of our analysis, having assumed for the sake of simplicity a Newtonian upper mantle. Strong evidence exists for a Non-Newtonian upper mantle rheology [148]. This would induce more complex upper mantle flow, as indicated by recent geodynamic models [149].

We must also acknowledge that our analysis for the sake of simplicity ignores lateral variations in asthenosphere thickness related to lithospheric thickness variations and the presence of cratonic keels. The latter would have important effects on the flow, particularly by reducing the extent of Poiseuille flow dominated area beneath these cratonic roots. Furthermore, we calculate the Poiseuille flow keeping the plume strength constant for the plumes considered. However, there are growing constraints on a time-dependent plume flux in the upper mantle, as indicated by numerous studies that attempt to estimate plume strength histories (e.g. [62,73,137,150]).

**Figure 11.**
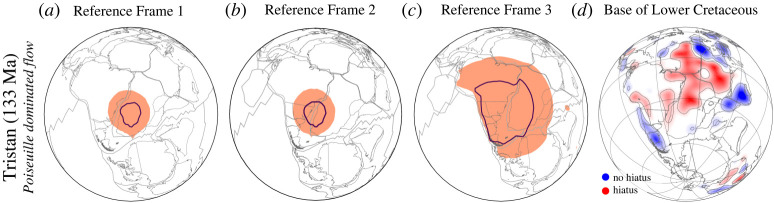
Estimates of Poiseuille flow dominated areas computed for Tristan at 133 Ma using three absolute plate motion models. Panel (*a*) is the same as figure 10 and assumes a global Mesozoic–Cenozoic plate motion model [27]. Panel (*b*) uses an Indo-Atlantic moving hotspot reference frame [151] for the past 100 Myrs and a TPW corrected paleomagnetic model [58] for older times. Panel (*c*) applies a reference frame of Indo-Atlantic moving hotspots [151] for the last 100 Myrs and of fixed African hotspots [155] for prior times. (*d*) BHS for Base of Lower Cretaceous (figure 3). Note that the size of hiatus and blank areas throughout Africa and South America in the Base of Lower Cretaceous map compares well to the extent of the Poiseuille-dominated area in Reference Frame 3. (Online version in colour.)

We close with some implications, starting with the Poiseuille flow dominated area $A_{f}$ computed for the Tristan hotspot (fifth row, fourth column figure 10) at 133 Ma. The area is approximately three times smaller than $A_{f}$ computed for the more recent Cenozoic plume events (fourth column figure 10). Moreover, the small size contrasts with the large hiatus mapped at the *Base of Lower Cretaceous*, figure 3*a*, for Africa and South America. This result is a consequence of the large Couette flow inferred at that time for the upper mantle beneath Africa and South America from the assumed plate motion model [27]. The latter is tied to a global moving hotspot reference frame [56] from present-day to 100 Ma and a TPW corrected paleomagnetic reconstruction [58] for times older than 100 Ma, which also includes a longitudinal shift of $10^{\circ }$ incorporated by Seton *et al.* [55]. O’Neil *et al.* [151] drew attention to the effects of absolute reference frames in past plate motion models, which may include TPW corrections and may assume fixed or moving hotspots or combinations of both. For times <80 Ma,  the reference frame differences are not discernible. But they grow further back in time. Moving hotspot reference frames have been computed from geodynamic models through so-called backward advection (e.g. [152]), which introduces uncertainties related to model parameters and starting conditions. It is possible that backward advection may mispredict Cretaceous hotspot motion, with consequences for plate motion reconstructions, and it is also possible that TPW corrections may overcompensate for the reorientation of the lithosphere-mantle system. Studies exist on absolute reference frame choices in geodynamic models (e.g. [153,154]). But further investigations and the use of mantle flow retrodictions (e.g. [142,143]) seem advised for improved assessments of past hotspot motion. The influence of absolute reference frame choices on the extent of Poiseuille-dominated area is explored in figure 11.

Next, we recall that Hayek *et al.* [45,46] interpreted hiatus maps in terms of the plate and plume mode of mantle convection (see [63] for a review of these convective modes). Broad conformable surfaces reveal the plate mode, while broad unconformable surfaces and areas of lack of signal express the plume mode. From the repeated appearance of continent-scale hiatus they deduced a significant role for the plume mode, which fits with geodynamic studies placing the total plume heat transport into the range of 10 TW (e.g.[156,157]), that is ∼20–30% of the global mantle heat budget (e.g. [158]). Our results suggest that plate motion variations should be included in this interpretation. Taking the current global RMS plate velocity of $5\;\mathrm{cm}\;{\mathrm{yr}}^{-1}$ [25] as representative and recalling that rapid plate motion variations in the range of $∼1\text{--}2\;\mathrm{cm}\;{\mathrm{yr}}^{-1}$ are potentially linked to the plume mode from our analysis, we speculate that ∼20–30% of overall plate velocities could be attributed to the plume mode.

Finally, we turn to plate boundary forces. Several studies emphasized their role in the Indian Ocean realm [115–118] owing to the topographic load of Tibet, as noted before. Others linked plate motion changes to Tibet’s evolving topography (e.g. [159,160]). This interpretation remains under debate [120]. But the significant contribution of topography, which is partially controlled by external processes such climate and erosion, to plate boundary forces may go some way in helping to explain why it remains difficult even with advanced mantle convection models, capable of generating plate-like surface motions, to reproduce the recent history of plate motions [161], as this would have to be parameterized in geodynamic simulations. Global coupled models of mantle and lithosphere dynamics may offer a possibility to address these challenges [114].

## 6. Conclusion

We have used continent-scale hiatus maps as a proxy for mantle flow induced dynamic topography and compared them with plate motion variations in the Atlantic and Indo-Australian realms since the Jurassic, building upon earlier work and exploiting growing observational constraints on both. We find that oceanic spreading rate changes and hiatus surfaces frequently correlate, except when plate boundary forces may play a significant role. Our work is geodynamically motivated from the description of asthenosphere flow beneath tectonic plates in terms of Poiseuille/Couette flow. This description explicitly relates plate motion changes, induced by evolving basal shear forces (Poiseuille flow), to non-isostatic vertical motion of the lithosphere. Our analysis reveals a timescale on the order of a geologic series between the occurrence of continent-scale hiatus and plate motion changes. It is best interpreted through dynamic topography response functions of dynamic Earth models, because a weak asthenosphere delays significant surface deflections into the final phase of material upwellings, when buoyant flow enters from the lower into the upper mantle. Our analysis suggests that the spatial scale of interregional hiatus, which is on the order of 2000–3000 km in diameter, should be interpreted through Poiseuille flow, where it corresponds to regions of active plume-driven upper mantle flow. We use fluid dynamic arguments to show that such active upper mantle flow can induce plate motion changes of $∼1\text{--}2\;\mathrm{cm}\;{\mathrm{yr}}^{-1}$, comparable to observations. Our results offer the motivation to further improve the temporal resolution of interregional geological maps, to enhance the constraints on past dynamic topography and associated paleogeography. This means such maps should be compiled more directly in relation to the relevant geodynamic processes that are revealed in the geological record. They also motivate one to pursue future studies of large-scale horizontal and vertical lithosphere motion in combination, as both of them track the expressions of past mantle flow. Such studies would provide powerful constraints for geodynamic inverse models of past mantle convection that are becoming feasible through the adjoint method.

## Data Availability

The oceanic spreading rates grid, the global plate motion model used to extract the spreading rates and the Base Hiatus Maps (BHS) have been previously published in Seton *et al.* [50], Müller *et al.* [27] and Hayek *et al.* [45,46], respectively.
